# The effect of universal maternal antenatal iron supplementation on neurodevelopment in offspring: a systematic review and meta-analysis

**DOI:** 10.1186/s12887-018-1118-7

**Published:** 2018-05-04

**Authors:** C. Jayasinghe, R. Polson, H. C. van Woerden, P. Wilson

**Affiliations:** 1 Ministry of Health, 385, Ven. Baddegama Wimalawansa Thero Mawatha, Colombo, 10 Sri Lanka; 2 Centre for Health Science, University of the Highland and Islands, Old Perth Road, Inverness, IV2 3JH UK; 3 Centre for Rural Health, University of Aberdeen, Old Perth Road, Inverness, IV2 3JH UK

**Keywords:** Iron supplementation, Antenatal, Offspring, Neurodevelopment, Iron deficiency anaemia

## Abstract

**Background:**

Although antenatal iron supplementation is beneficial to mothers, its impact on the neurodevelopment of offspring is controversial. A systematic review and meta-analysis was undertaken to assess whether routine maternal antenatal iron supplementation confers later neurodevelopmental benefit to offspring.

**Methods:**

Electronic databases were searched using MESH terms or key words and identified papers were reviewed by two independent reviewers. The study quality was assessed using the Cochrane risk of bias assessment tool. The review was registered in the PROSPERO CRD data base.

**Results:**

Seven publications were identified, based on four randomised trials published between 2006 and 2016. Three of the trials were in the Asian sub-continent. A range of tools were used to evaluate neurodevelopment. Meta-analysis of outcomes from the three RCTs meeting our inclusion criteria showed minimal effect of antenatal iron supplementation on the neurodevelopment of offspring, which was not statistically significant: weighted mean difference of 0.54 (95% CI: -0.67 to 1.75); test for overall effect Z = 0.87; *p* = 0.38; and heterogeneity 48%. Meta-analysis of outcomes of these RCTs at later stages of development produced similar results.

**Conclusions:**

The benefit of routine antenatal iron supplementation on neurodevelopment in offspring was not statistically significant in this relatively limited set of trials, and some benefit cannot be excluded in areas with a high prevalence of maternal anaemia. A large randomized controlled trial showing significant benefit would be required to modify our conclusions.

**Electronic supplementary material:**

The online version of this article (10.1186/s12887-018-1118-7) contains supplementary material, which is available to authorized users.

## Background

Iron supplementation during pregnancy is routinely provided in many countries. The WHO currently recommends that 30–60 mg of elemental iron is given daily from as early as possible during pregnancy [1]. This public health policy aims to improve pregnancy outcomes and to reduce maternal anaemia [2, 3]. The beneficial effect of iron supplementation during the antenatal period in relation to birth weight, physical growth and perinatal mortality has been evaluated in several studies [2, 4]. An association has been shown between poor performance in mental and psychomotor tests and lower iron status in utero [5]. A randomized trial conducted in Bangladesh supplementing iron in various doses, and providing multiple micronutrients in food items for antenatal mothers, did not shown any significant effect on motor development of offspring at seven months old [6]. However, a finding of the northern Finnish birth cohort of 1966 was that low maternal haemoglobin levels in the final stage of pregnancy was linked to poorer educational achievement of offspring [7]. Another study showed that antenatal iron and folic acid supplementation reduced the prevalence of anaemia from 67% to 38.4%, and that there was an inverted U shaped relationship between maternal haemoglobin concentration and motor function of one year old children [8]. Antenatal supplementation with multiple micro-nutrients including iron and folic acid has been shown to improve motor and cognitive function of offspring at 3.5 years more effectively than supplementation with iron and folic acid alone [9]. These studies give rise to the question as to whether antenatal iron supplementation on its own leads to better neurodevelopment in offspring, which is the focus of this review.

The uncertainty in current evidence has been recognised by previous researchers who have recommended that the long term effects on child development of iron supplementation during pregnancy should be assessed as an area of priority [10–12]. The last available review of the effect of iron supplementation in non-anaemic pregnant women, infants and young children on mental performance and psychomotor development of children was only able to include one randomised controlled trial (RCT), which was undertaken in Australia and was inconclusive [13]. We have therefore undertaken a systematic review of the literature to examine the effect of iron supplementation in pregnancy on neurodevelopment in offspring. Neurodevelopment has been defined in this context to include aspects of behaviour, cognitive development, mental development or intellectual development.

## Methods

### Search strategy

Electronic databases, namely Ovid Medline, EMBASE, CINAHL, SCOPUS, Cochrane Central Register of Controlled Trials (CENTRAL), and Web of Science were searched between 5th January and 10th February 2016. The review included randomised controlled trials and cohort studies reporting on the routine use of iron supplementation during pregnancy and associated assessment of child development as an outcome. The inclusion of cohort studies is in line with best practice where published evidence is very sparse [14]. Non-randomized observational studies were not included to minimize the risk of selection bias and confounding (we found no such studies in our literature searches in any case). Both medical subject headings and free text keywords were utilised as search terms. In this review, synonyms for the term ‘child development’ included ‘children’s brain development’, ‘IQ’, ‘cognitive, psychomotor or behavioural development’ (See search criteria in the Additional file 1). Although we planned to include cohort studies and of iron supplementation in pregnancy followed by assessment of child development, in addition to RCTs, none fulfilled the inclusion criteria.

Papers reporting the outcome of interest using any type of neurodevelopmental assessment tools were accepted for the review. The references in the identified articles and in related review articles were manually searched for additional literature that might otherwise have been missed by the electronic databases. The review included only original research articles published as full text in the English language and conducted on human beings.

### Inclusion criteria

The inclusion criteria were: RCTs and cohort studies, reporting the association between iron supplementation during pregnancy and neurodevelopment in offspring undertake before the offspring had reached puberty, in practice, 12 years of age. Iron supplementation had to be the key difference between at least one intervention group and one control group.

**PICO** [15, 16] **definitions:**

- **Population:** Pregnant women
- **Intervention**: Iron supplementation (or iron with a micronutrient, where the comparator was the micronutrient alone)
- **Comparator**: Placebo supplementation, or no supplementation, or micronutrient supplementation but without iron or ferrous compounds
- **Outcome**: Child neurodevelopment, including mental development, cognitive function, psychomotor development, IQ, behaviour or other aspects of neurodevelopment

### Study selection

**Fig. 1:**
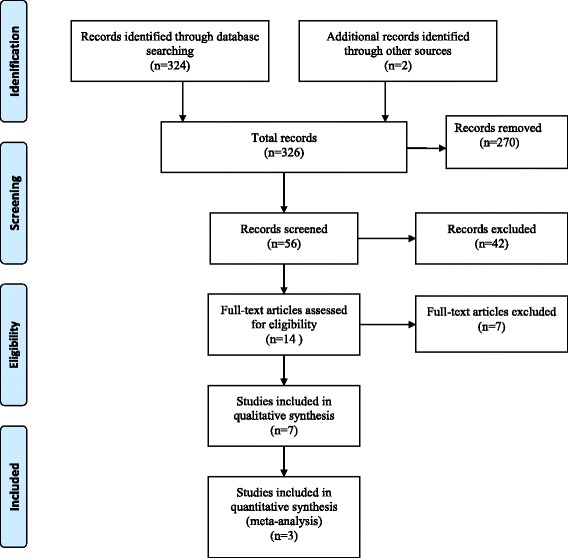
PRISMA Flow diagram for identified studies

Titles and abstracts retrieved from the search were jointly assessed by two reviewers (JC & HvW) for eligibility by reading titles and, where appropriate, abstracts. Studies that were obviously irrelevant were excluded. A study selection flow diagram is shown in Fig. 1 based on the PRISMA tool [17]. The full text of potentially relevant remaining articles was assessed by one reviewer, with review by a second person, to establish final eligibility. An update of the literature search was carried out to 30th July 2016. One study published in May 2016 was found and was included in the review [18].

### Data extraction and quality review process

**Fig. 2:**
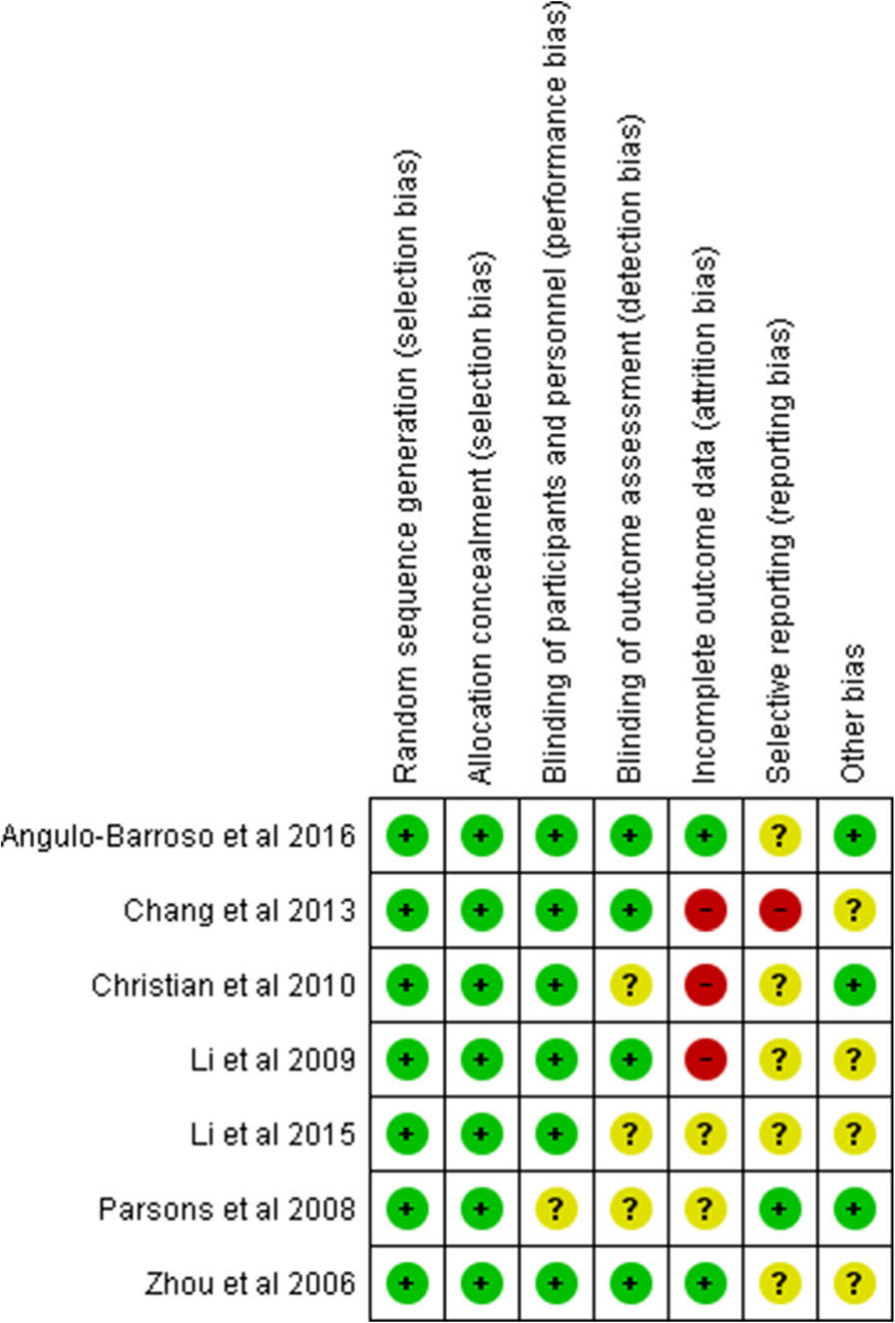
Risk of Bias Assessment

Data from the included studies are summarized in Table 1. A data extraction instrument designed in line with Cochrane data extraction sheets was used [19], which included the author, title, journal, year of publication, study setting, study design, study population, sample size, participants’ characteristics, country where the research was carried out, type of iron supplementation, definition of iron deficiency anaemia, the method or tool used to assess child development, follow up period and study outcomes relevant to the review. Key data from each of the selected studies were at first reviewed independently by two reviewers (JC & HvW) and then jointly for quality assessment using the Cochrane Risk of Bias check list [2]. Discrepancies were forwarded for arbitration to the third reviewer (PW). We had only two disagreements regarding the blindness of intervention group of the participants in Parsons et el. [20] and inclusion of a Mendelian randomization study for the review [21]. Risk of bias was rated as high, low or unclear (see Fig. 2).

**Table 1:** Summary of papers identified

Author	Study design	Title of article	Population(Location) and study setting	Year of enrolment	Original number enrolled and number followed up	Control and intervention	Primary outcome and tools used	Age of the children when outcome measured
1. Zhou et al. 2006 [11]	RCT	Effect of iron supplementation during pregnancy on the intelligent quotient and behaviour of children at 4 years of age: long term follow up of a randomized controlled trial	AMBIT Women’s and children’s hospital, Adelaide, Australia Urban setting	Original trial 1997–1999 May 2002 Jan 2004	430 pregnant women enrolled IQ assessed I -153 C -149 Behaviour assessed I -151 C -149	I = from 20wks of gestation to delivery birth 20 mg of iron C = placebo	Stanford Binet Intelligence Scale (SBIS) used to assess IQ and Strengths and Difficulties Questionnaire (SDQ) used to assess behaviour	4 years
2. Parsons et al. 2007 [20]	RCT	Effect of iron supplementation during pregnancy on the behaviour of children at early school age: long term follow up of a randomised control trial	Ditto	April to Nov 2006	430 pregnant women enrolled Behaviour assessed 264 *I* = 132 C - 132	Ditto	Strength and Difficulties Questionnaire (SDQ) used to assess Behaviour	6–8 years
3. Li et al. 2009 [27]	Follow up of RCT	Effect of maternal multi-micronutrient supplementation on the mental development infants in rural Western China	China Rural setting of Shaanxi Province of Western China	Original research Aug 2002 to Jan 2006 Followed up from Jan 2004- Dec 2004	5828 women enrolled for the original trial and there were 4604 live births. For this component 1305 subset of infants assessed I – 471&396 C - 438	Once pregnancy diagnosed but before 28 weeks of gestation I = FA 400+ Fe60 C = FA 400	Bayle’s Scale of Infant development (BSID) used to assessed Mental & psychomotor development	3, 6 & 12 month of age
4. Chang et al. 2013 [26]		Effect of iron deficiency anaemia in pregnancy on child mental development in rural China	Ditto	2004–2006	5828 women enrolled for the original trial and there were 4604 live births. For this component 1286 subset of children assessed C = 468 I-423 & 395	I = FA 400 + Fe60 C = FA 400	Bayley’s Scale of Infant development (BSID) used to assessed Mental Development Index and Psychomotor Development Index	3, 6, 12, 18, & 24 months of age
5. Li et al. 2015 [28]	Follow up of RCT	Prenatal micronutrient supplementation is not associated with intellectual development of young school aged children	Ditto	2012–2013	For this component subset of 1744 children were assessed C -604 I -562 & 578	I = FA 400+ Fe60. C = FA 400	WISC-IV (Wechsler intelligence scale for Children Verbal comprehension scale, working memory index, perceptual reasoning, processing speed index	7–9 years
6. Christian et al. 2010 [12]	Follow up of RCT	Prenatal micronutrient supplementation and intellectual and motor function in early school aged children in Nepal	Nepal Rural setting of Southern Sarlahi district.	Original trial done from 1999 to 2001. Follow-up study from June 2007–April 2009	4998 women enrolled for the original and for this component 676 infants were enrolled. C -177. I -103 &178 7218	From 11 weeks of gestation I = Fe60 + FA 60 + Vitamin A. C=Vitamin A	Universal Non Verbal Intelligence Test (UNVIT)	7–9 years of age
7. Angulo-Barroso et al. 2016 [18]	RCT	Iron supplementation in pregnancy or infancy and motor development: a RCT	China Rural setting of Hebei province.	June 2009 to Dec 2011. assessment done from Sep 2010 to Mar 2013	2371 recruited for the original study. I-1185. C- 1186	From enrolment to birth I = Fe300 + FA. C = Placebo + FA	Peabody Development Motor Scale second edition PDMS II). Used to assess gross motor development	9 months of age

### Analysis

A meta-analysis was undertaken using Review Manager 5.3 [22]. Studies were excluded from the meta-analysis if they included the same participants who had been reported in another included paper. Where several studies followed up the same group of participants, the study entered into the meta-analysis was that with the length of follow up that was closest to other included studies. Forest plots were created using Review Manager and weighted mean differences were calculated.

A random effects model was used, as there was a potential for heterogeneity arising out of the range of assessment methods used by the included studies. Under such circumstances this is more robust than using a fixed effects model. The review was registered in the PROSPERO CRD data base, registration number CRD 42016037114.

## Results

A total of 324 articles were identified by searching electronic databases and a further two were identified by manual bibliographic searches (Fig. 1). Of those, 270 articles were excluded during initial screening because they did not report the effect of iron supplementation on child neurodevelopment. The full texts of the remaining 56 articles were again reviewed to assess whether their research objectives met our inclusion criteria. This full text review led to exclusion of a further 42 articles as the outcome of interest was outside the scope of our objectives. Hence, 15 articles were selected by both reviewers, one of which was excluded because the intervention included both iron and folic acid [12]. Six articles were excluded as they assessed different doses of antenatal iron but did not utilise a control group who did not receive a supplement [6–9, 23, 24]. One Mendelian randomization study was removed because final analysis was undertaken according to the genetic variant and the effect of iron supplementation or non-supplementation could not be established [21]. The remaining seven articles were included in the qualitative synthesis (Fig. 1).

### Study characteristics

The seven papers we reviewed were based on four trials and were published between 2006 and 2016 (Table 1). Six papers were extensions of three original RCTs. Sample size varied from 264 to 1714. The reviewed papers are summarised in Table 2 and in the Supplementary file. They included pregnant women from three different countries: China, Australia and Nepal.

**Table 2:** Summary of parameters for the intervention and control groups for the key studies

Authors	Intervention group			Control group		
	Mean	SD	N	Mean	SD	N
Zhou et al. 2006 [11]	109	11	153	109	11	149
Parsons et al. 2008 [20]	8.6	6.3	132	9.2	5.9	132
Li et al. 2009 [27]	102.44	4.475	438	102.65	4.42	471
Chang et al. 2013 [26]	100.2	15.7	466	98	15.3	384
Li et al. 2015 [28]	88.98	12.69	562	89.82	14.07	604
Christian et al. 2010 [12]	51.7	8.5	103	48.2	10.2	177
Angulo Barroso et al. 2016 [18]	87.5	0.95	298	87.4	0.95	288

There was variation in the dose of iron supplementation and the gestation period at which supplementation was initiated. Maternal iron supplementation varied from 20 mg daily up to 300 mg daily (Table 1). Initiation of iron supplementation varied from 11 weeks gestation to 20 weeks gestation. Two RCTs were carried out in areas where anaemia prevalence was high and one in an area where it was low [11, 20, 25].

Three publications [26–28] were follow up studies of a single RCT [29], with outcomes measured at different stages in the development in the offspring, where mothers had received iron supplementation. Similarly, another two publications [11, 20] were based on another RCT where mothers had received iron supplementation, with outcomes measured at different stages in the development in the offspring [25]. We excluded the study carried out in Nepal for the meta-analysis [12]. This was because in the original study there was a control group receiving folic acid only supplementation, but this group was not included in the follow up study [30]. In the trial by Angulo Barroso et al., although there were four arms, for this review we only considered the outcomes related to the arm that received iron supplementation during pregnancy and the control group [18]. The children who received iron supplementation after birth were not included in our analysis.

### Anaemia prevalence

The prevalence of anaemia varied markedly across the countries included in this review. A study linked to the RCT in China gave a prevalence of anaemia undertaken in the third trimester of gestation of 57% [29]. In Nepal the baseline prevalence of anaemia in the study area was more than 40% [30, 31]. In contrast the prevalence of iron deficiency anaemia among those recruited in Australia was 11% [25]. The estimated global prevalence of anaemia in the general population is 24.8%, with an estimated anaemia prevalence in preschool children of 47.4%, in pregnant women 41.8% and in non-pregnant women 30.2% [32].

### Quality of reporting

The quality of each of the studies was assessed according to the Cochrane Risk of Bias tool. All the papers were generally acceptable for most items in the quality assessment matrix [19]. All studies used random sequence generation and allocation concealment. In all, except one paper [20] participants were blinded. The extent of blinding of outcome assessment was unclear in three studies [12, 20, 28]. There was very high loss to follow up in three papers [12, 26, 27] and follow up rates were unclear in two papers (Fig. 2). Analysis of primary outcome data was undertaken on an ‘intention to treat’ basis in all except one study [26].

### Mental development outcome

Different scales of measurement were used in different studies, namely: Bayley’s Scale of Infant Development (BSID); Stanford-Binet Intelligence Scale (SBIS); Strengths and Difficulties Questionnaire (SDQ); Wechsler Intelligence Scale for Children (WISC); Universal Nonverbal Intelligence Test (UNVIT) and the Peabody Developmental Motor Scale (PDMS). Although not initially designed for that purpose, the SDQ has been used for studies to assess language, social, emotional problems and as a neurodevelopment assessment tool at younger ages, and was therefore considered a valid tool [33]. All the other tools have been used to assess intelligence and cognitive function in children [34].

### Effect of intervention

**Fig. 3:**
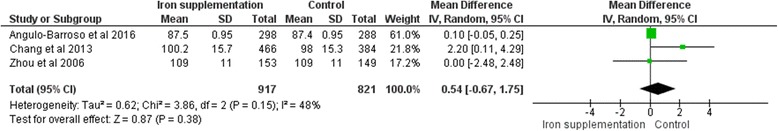
Forest plot of three RCTs included in a meta-analysis

Three publications were not included in the meta-analysis as they used the same participants at different ages [20, 27, 28]. The RCT carried out in Nepal [12] was not included as the intervention group was supplemented with both iron and folic acid. Three RCTs were included in the meta-analysis and provided a weighted mean difference of 0.54 units (95% CI -0.67 to 1.75), with an overall effect size z = 0.87 (*p* = 0.38) and a heterogeneity of 48% (*p* = 0.15). The level of heterogeneity was moderate (I^2^ = 48%) and not statistically significant (Fig. 3). The results suggest that routine (as opposed to targeted) maternal iron supplementation does not have a statistically significant effect on later neurodevelopment in offspring.

## Discussion

The primary objective of this systematic review was to determine whether antenatal iron supplementation provided any neurodevelopment benefit for offspring. Among the seven eligible publications reviewed, only one study was found that primarily focused on iron supplementation in pregnancy and motor development of offspring [18]. The other six publications were based on RCTs where the original focus was on different objectives and extensions of the initial trials were undertaken to examine child neurodevelopmental effects. Our conclusion is that any effect of routine iron supplementation on its own is likely to be very small.

We would suggest that the benefits of routine antenatal supplementation are likely to be related to the underlying prevalence of anaemia in a given population. The greatest effect was observed where anaemia prevalence was highest [26] and the least effect was observed where anaemia prevalence was lowest [11].

There is evidence that the global prevalence of anaemia in pregnancy is falling, as a study carried out to estimate the global mean haemoglobin concentration among women of reproductive age (15–49 years) has shown that anaemia prevalence decreased from 43% to 38% in pregnant women from 1995 to 2011 [35]. This study suggests that in countries where the prevalence of anaemia is low or falling, there may be a case for limiting antenatal prescription of iron to women who are anaemic and have low iron stores.

An outstanding question, which merits further research, is whether iron supplementation is best seen as one of a cluster of micronutrients that need to be available during foetal growth to maximise neurodevelopment, as two studies have provided some evidence to support antenatal iron supplementation when provided in conjunction with other micronutrients. In one study carried out in Nepal, iron and folic acid supplementation improved mean Universal Nonverbal Intelligence Test scores on psychometric testing compared to a control group [12]. A study in China also demonstrated a small improvement in mental development raw scores at one year of age in an iron supplementation group, compared to a non-supplementation group [26]. Another study showed a lower mental development index in children born to mothers with iron deficiency anaemia during pregnancy compared to those whose mothers were not iron deficient during pregnancy [27]. As previously stated, this may reflect the interplay between the availability of iron and other aetiological factors.

Although not include it in the meta analysis, a study carried out in rural Vietnam demonstrated significant improvement in infant cognitive outcomes at six months of age following twice weekly antenatal iron supplements rather than daily supplements [36]. In an Indonesian trial, supplementation with daily iron, weekly iron with Vitamin A, and weekly iron alone was provided from 18 weeks of gestation until delivery followed by assessment of cognitive and psychomotor development of infants. The infants of all three supplemented groups had similar mental and psychomotor development indices at six and twelve months of age [25].

It is important to consider whether routine iron supplementation could have adverse, as well as beneficial effects, as the possibility exists of adverse effects from excessive iron supplementation in those who are not iron deficient. Abnormal teacher rated peer relationship problem subscale scores in the Strengths and Difficulties Questionnaire were associated with iron supplementation in an Australian study (RR = 3.70; 95% CI 1.06 to 12.91; *p* = 0.026) [20]. In this study, the attrition rate was almost 50% (only 112 out of 216 who were initially randomized completed the study) and the results may have been biased by differential drop out of healthy children. The findings of this study therefore need interpreted with caution. In summary, the meta-analysis of three RCTs did not show a statistically significant benefit from routine antenatal iron supplementation on the neurodevelopment of offspring: weighted mean difference of 0.54 units (95% CI 0.67 to 1.75).

### Strengths and limitations

This review included RCTs without limiting the date of publication. It provides a substantial update on a previous review, which had slightly different aims, but only identified a single RCT [13]. The assessment of quality in the RCTs was based on the methods described in the original trials, bearing in mind that the original studies were conducted with different objectives in some cases. The tools used to assess the mental development of children varied widely, as did the neurodevelopmental domains that were evaluated, and some tools had not been validated in the language into which they had been translated.

A limitation of our study is that we may have missed unpublished data or studies published in the grey literature. We may also have missed studies that were not published in English. The majority of the included trials were performed in Far Eastern countries and the generalizability to other areas in the world requires some caution.

The most appropriate follow up period for a study such as this is difficult to determine. Assessment a week after birth would clearly provide an inadequate follow up period and follow up after the age of 12 years is probably affected by a wide range of other environmental factors including puberty. Follow up at younger ages might reduce heterogeneity, as most studies undertake follow up within the first five years. However, follow up at older ages is probably a better proxy for identifying lifelong impacts of antenatal iron supplementation. A case can be made for either approach. Sensitivity analysis around age (not shown) did not indicate significant differences in the outcome of the meta-analysis when the age at which assessment was undertaken was varied. In general, neurodevelopmental deficits present in early life will be continued to some extent in later years so the direction of any effects should be the same regardless of age or precise developmental assessment method.

Assessment of neurodevelopmental function is challenging. There is no universally recognized method of assessing such development, and as a result different studies inevitably use different tools. Stanford Binet Intelligence Scale is used to assess cognitive ability and intelligence, Bayley’s Scale of Infant Development is used for cognitive, language, motor, social and emotional assessment and the Peabody development scale is used for fine and gross motor development assessment. Restricting our analysis to only one dimension of neuro-development such as cognitive function might have missed wider effects, but does introduce conceptual heterogeneity.

It could be argued that this review should be restricted to a descriptive analysis of the papers that have been identified, on the basis of the limited and diverse nature of the assessment methods that were used. However, quantification of results does add value, even when this needs to be presented with appropriate caveats. On balance, we believe that the advantages of including a range of studies in our meta-analysis outweigh potential disadvantages and a meta-analysis has therefore been provided.

### Implication for research

It is possible to speculate that one micronutrient deficiency is often associated with the presence of other micronutrient deficiencies. As a result, future research might assess whether larger benefits can be obtained by the use of enriched diets with high multi-micronutrient content, or multi-micronutrient supplementation, which would address deficits across a wider range of enzymatic pathways and potentially have a greater overall effect on neurodevelopment in the foetal brain.

## Conclusion

We have attempted to establish whether there are positive neurodevelopmental outcomes from antenatal iron supplementation and our conclusion is that there was little or no evidence of benefit in routine use. Any effect appears to be small. We consider that a large randomized controlled trial of universal iron supplementation would be required to substantially change our conclusions.

## Additional file

Additional file 1: Part 1: Demonstration of search strategy for Medline; Part 2: Summary of the selected studies. (DOCX 24 kb)

## Abbreviations

BSID: Bayley’s Scale of Infant Development
IQ: Intelligence Quotient
MESH: Medical Subject Headings
PDMS: Peabody Developmental Motor Scale
PICO: Population, Intervention, Comparator, Outcome
PRISMA: Preferred Reporting Items for Systematic Reviews and Meta-Analyses
RCT: Randomised controlled trial
SBIS: Stanford-Binet Intelligence Scale
SDQ: Strengths and Difficulties Questionnaire
UNVIT: Universal Nonverbal Intelligence Test
WISC: Wechsler Intelligence Scale for Children
