# Supplementary material for: The effect of universal maternal antenatal iron supplementation on neurodevelopment in offspring: a systematic review and meta-analysis
Source: BMC Pediatr. 2018 May 4;18:150. doi: 10.1186/s12887-018-1118-7 (PMC5936025; doi:10.1186/s12887-018-1118-7)
Supplement: Supplementary file 1 — Part 1: Demonstration of search strategy for Medline; Part 2: Summary of the selected studies. (DOCX 24 kb) [file 12887_2018_1118_MOESM1_ESM.docx]

**Additional file**

**Supplementary Information**

**Part 1: Demonstration of search strategy for Medline (Ovid format)**

| **No** | **Search terms** |
| --- | --- |
| **1** | **Anaemia, Iron deficiency** |
| **2** | **Iron** |
| **3** | **Haemoglobin** |
| **4** | **Dietary Supplements** |
| **5** | **Ferrous Compounds** |
| **6** | **1 or 2 or 3 or 4 or 5** |
| **7** | **Pregnancy** |
| **8** | **Prenatal care** |
| **9** | **7 or 8** |
| **10** | **Child development** |
| **11** | **Child cognition** |
| **12** | **Child brain development** |
| **13** | **10 or 11 or 12** |
| **14** | **Randomized control trials** |
| **15** | **Clinical trials** |
| **16** | **Cohort studies** |
| **17** | **Follow up studies** |
| **18** | **14 or 15 or 16 or 17** |
| **19** | **6 and 9 and 13 and 18** |
| **20** | **Limit 19 to(English language and humans)** |

**Part 2: Summary of the selected studies**

**Zhou *et al.* 2006**

This was a double blind randomized control trial conducted in Australia by recruiting 430 antenatal mothers who received care at the Women’s and Children’s hospital Adelaide^,^ Australia during 1997-1999. Participants were given either 20 mg/d of Iron or placebo from 20 weeks gestation. Childhood Intelligence Quotient (IQ) has been assessed by using the Stanford-Binet Intelligence Scale at 4 years of age. Childhood behavior was assessed with the parent version of the Strengths and Difficulties Questionnaire (SDQ). Out of 430 pregnant women, 216 were assigned to receive iron whereas 214 were assigned to receive placebo. There were 219 children in the intervention group and 214 children in the placebo group. Finally 153 and 151 children were included for IQ and behavior analysis from the iron supplemented group. From the placebo group 149 children were included for IQ and 149 for behavior analysis. The mean IQ was not significantly different (p=0.980) between the children of iron supplemented mothers (109 +/- 11; n=153) and the children of the mothers in the placebo group (109 +/-; n=149).

**Parson *et al.* 2008**

The same mothers and children included in the Zhou et al 2006 study were followed up for another four years until children were age 6-8 years. Children’s behavior was assessed by using parent and teacher rated version of Strength and Difficulties Questionnaire (SDQ). Altogether 264 (61% of the total) were assessed at the follow up. From the intervention group 132 were included in parent analysis and 112 were included in teacher analysis whereas 132 were included in parent analysis and 113 were included in teacher analysis from the placebo group. The mean score of parent rated and teacher rated SDQ scores did not differ between iron-supplemented and placebo groups. There was a higher incidence of abnormal teacher rated peer problems score in the intervention group than the placebo group [RR=3.70, (1.06-12.91; p=0.026)].

**Li *et al.* 2009**

This is a follow up study of Zeng et al (2008) conducted in two poor rural counties in North West China from Aug 2002 to Jan 2006 (Zeng et al 2008). This was a double blind cluster randomized control trial. The villages were randomly assigned to the three supplementation groups, daily folic acid, folic acid plus iron and multi-micronutrient (MMN) supplements. For the total period of trial 5828 pregnant women were recruited from the 531 villages and 4604 single live birth were reported. Singleton births between Jan 2004 and Dec 2004 were recruited for the analysis of MD (mental development) raw score and PD (psychomotor development) raw score. Finally 471 from the folic acid group, 438 from the folic acid plus iron group and 396 from the MMN groups were analyzed at 3,6,12 months of age by using Bayley Scales of Infant Development (BSID). A four level analysis was developed to compare the MD and PD raw score.

**Chang *et al.* 2013**

This is a follow-up study of Zeng et al (2008) conducted in two rural counties in North West China from Aug 2002 to Jan 2006. Assessment of mental, psychomotor development of children was conducted at 3, 6, 12, 18 and 24 months of age by using BSID. A total of 1286 women with singleton full term births had attended this followed up study. Finally 850 women and the children met the inclusion criteria. Among those 468 have been from the folic acid group, 423 from the iron plus folic acid group and 395 from the MMN group. At 18 months of age the differences in MDI between the prenatal IDA group and the prenatal non IDA were only present in the folic acid and MMN supplied group and not in the iron supplemented group. Adjusted difference [5.1 (1.2-9.0; p=0.010)].

**Li *et al.* 2015**

This is also follow-up study of Zeng et al 2008. For this component 1744 children were assessed for intellectual function at aged 7-9 years by using Wechsler Intelligence Scale for Children (WISC-IV). 604 from folic acid group, 562 from folic plus iron group and 578 from MMN supplied group were analyzed. The mean differences were not significant between prenatal folic acid supplementation and either the iron and folic acid supplement or MMN supplemented groups after adjusting for confounders.

**Christian *et al.* 2010**

This is a cluster randomized double blind controlled trial carried out in rural village in Nepal between 1999 and 2001. These children were assessed at age 7-9 years in 2007 to 2009. In 2000 to 2001 there were 3351 live births. There were 879 births in the control group and the control group was only supplied with Vit A. All other intervention groups also supplied Vit A. There were 773 in the iron folic acid group, 827 in the iron, folic acid and zinc group and 872 in the MMN group. Children’s intellectual function was assessed using the Universal Nonverbal Intelligence Test (UNIT). The numbers of children were 177 from the control group and 103 from the iron plus folic acid group. This study showed that maternal prenatal supplementation with iron and folic acid was positively associated with general intellectual ability in some aspects of motor function.

**Angulo Barroso *et al.* 2016**

This was a RCT conducted in rural China in June 2009 to Dec 2011. This was a combination of pregnancy iron supplementation RCT with the infant iron supplementation RCT. Total of 2371 mothers were enrolled for the pregnancy RCT and out of those 1185 were in the iron-supplemented group and 1186 were in the placebo group. For the infant RCT, 730 were enrolled from the placebo group and 752 from the iron supplemented group. Gross motor assessment had been done at 9 months of age for 312 from the placebo group and 327 from the intervention group using the Peabody Developmental Motor Scale (PDMS). Result showed that iron supplementation in infancy with or without iron supplementation in pregnancy improved gross motor test score.
